# Denervation pseudo hypertrophy of the calf: An important cause of lower limb swelling

**DOI:** 10.1016/j.radcr.2022.02.066

**Published:** 2022-03-22

**Authors:** John P. Hynes, David Glynn, Stephen J. Eustace

**Affiliations:** a Department of Radiology, National Orthopedic, Hospital of Ireland, Dublin, Ireland; b Department of Radiology, University College, Dublin, Ireland

**Keywords:** Denervation pseudohypertrophy, Calf swelling, Weightlifting, Sports imaging

## Abstract

Denervation pseudohypertrophy is an uncommon cause of limb swelling, which may be overlooked. It is an important diagnosis to arrive at, as it instructs the search for an underlying cause which may itself require intervention. We present the case of a 32-year-old male rugby player with a 2-year history of left calf swelling and intermittent pain and tightness. He described a previous history of 2 left sided lumbar micro-discectomy surgeries. There was no tenderness or sensory deficit on examination. MRI of the left calf revealed muscular enlargement, with fat interspersed between the muscle fibers, in keeping with pseudohypertrophy. This has a number of causes, in this cause attributed to lumbar radiculopathy. This case highlights a rare but important cause of limb swelling which should be considered in the workup of a unilateral swollen limb.

## Introduction

Muscle denervation pseudohypertrophy can be seen in a variety of conditions and settings, including secondary to peripheral nerve traumatic injury, peripheral neuropathic conditions (classically diabetes mellitus), disorders of the anterior horn cells such as poliomyelitis, and due to radiculopathy [1]. The typical magnetic resonance (MR) imaging appearances of muscle denervation are well described, with muscle oedema seen in the acute setting followed in most instances by atrophy, and loss of muscle volume [2]. More rarely, pseudohypertrophy may occur whereby the muscle fibers are enlarged due to the accumulation of fat. We describe a case of a 32-year-old rugby player with a history of degenerative lumbar disk disease requiring discectomy who presented with a swollen left calf due to muscular pseudohypertrophy.

## Case report

A 32-year-old male rugby player presented with a 2-year history of swelling in the left calf. He described intermittent pain in the left calf with a sensation of tightness and occasional tingling in the left foot. Past medical and surgical history was significant for previous lumbar microdiscectomy.

On examination there was asymmetry of the calves, the left being larger than the right. There was no tenderness on palpation. Pulses were present and normal. There was no sensory deficit.

**Fig. 1:**
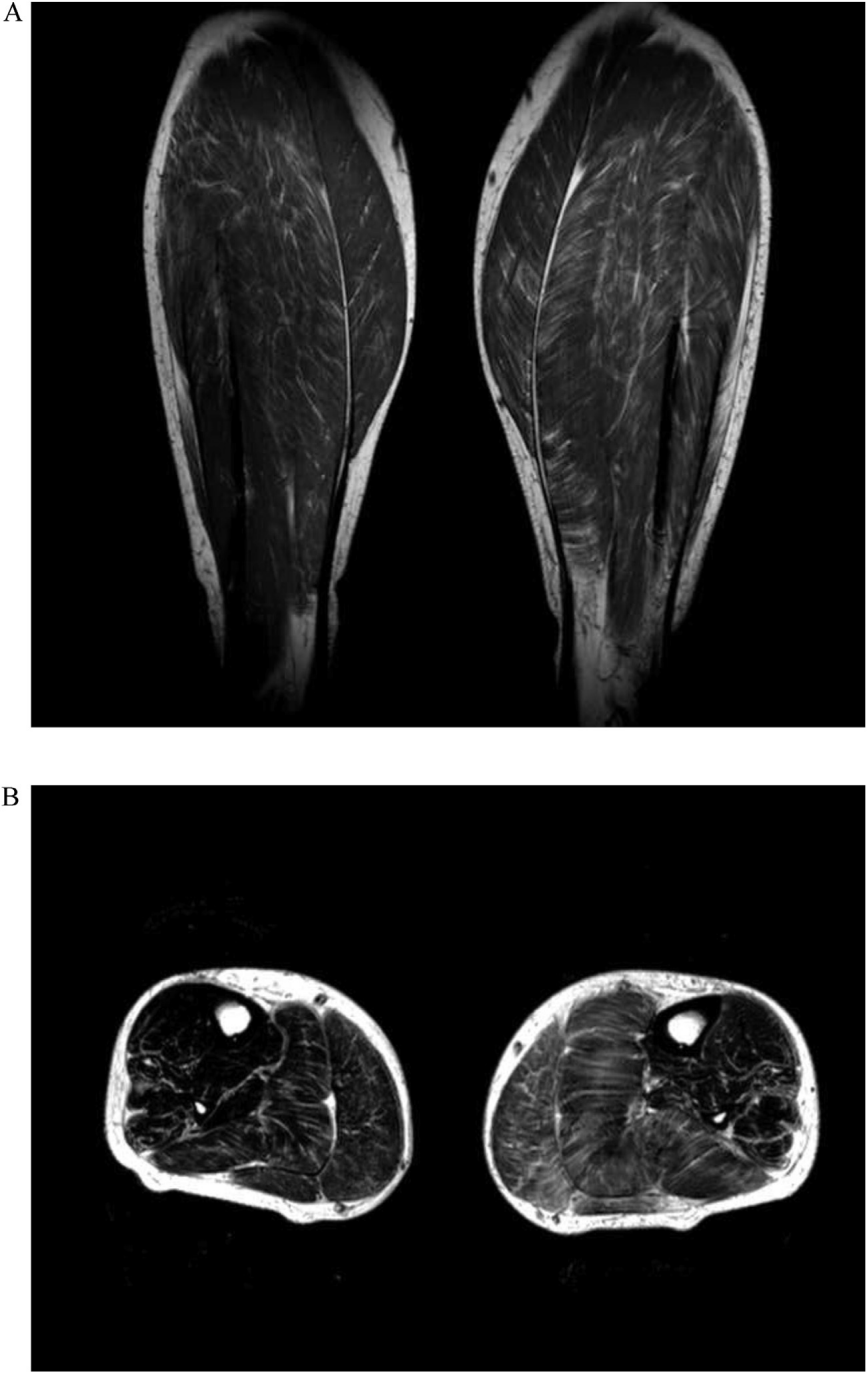
Coronal T1-weighted (Fig. 1A) and Axial T2-weighted (Fig.1B) images of the calves demonstrating asymmetric enlargement of the musculature of the left calf with evidence of fatty infiltration.

The patient was referred for compartment pressure testing, which was normal. An MRI scan of the calves was subsequently performed. This demonstrated asymmetric enlargement of the musculature of the left calf with evidence of fatty infiltration (Fig. 1). The appearances were most marked in the superficial posterior compartment, and were felt to be suggestive of denervation pseudohypertrophy.

Ultrasound guided muscle biopsy of the left gastrocnemius muscle was then performed. Histopathological analysis demonstrated atrophy of skeletal muscle fibers, with extensive infiltration by mature adipose tissue. These findings, in concordance with the clinical history and the appearances at MRI were felt consistent with partial denervation pseudohypertrophy of the calf musculature reflecting incomplete neural injury due to degenerative lumbar disc disease and possibly also contributed to by post-operative scarring.

## Discussion

Denervation pseudohypertrophy is an uncommon and therefore possibly overlooked cause of unilateral calf swelling. It is characterized by muscular enlargement due to the accumulation of fat between the muscle fibers. There is also muscular enlargement in true hypertrophy, however the enlarged muscle would demonstrate normal homogenous muscle signal intensity. Denervation pseudohypertrophy occurs due to partial denervation and has been described in association with a variety of conditions including diabetic neuropathy [1], peripheral nerve injury [3], and spina bifida [1]. It has also previously been described in the setting of radiculopathy [4] as in the case outlined here, although there is a relative paucity of evidence in the literature with only case reports, and small case series described. It is difficult to extrapolate an accurate numerical estimate of its prevalence in the population, and it is likely that the prevalence is underestimated.

The diagnosis of muscular hypertrophy requires multidisciplinary input as outlined in our case, with good clinical history and examination, evaluation with MRI and histopathologic analysis all necessary. An accurate diagnosis is important as it instructs additional investigations as required to elucidate an underlying cause [6].

Deep infiltrating intramuscular lipoma is an important differential consideration [1]. This may be difficult to distinguish from pseudohypertrophy on the basis of imaging alone and histopathological analysis may be required. Dystrophic muscular conditions, most commonly Duchenne muscular dystrophy, can cause pseudohypertrophy of the calves. Typically this will be bilateral, beginning in early childhood with progressive involvement of additional muscular groups [5]. The differential diagnosis of a unilateral swollen calf also includes vascular etiologies, particularly deep venous thrombosis, as well as infection, and tumor. Rarely, congenital conditions such as Klippel-Trenauny or Proteus [7] syndromes can cause limb enlargement.

There are no descriptions in the literature to our knowledge of successful interventions for denervation pseudohypertrophy secondary to lumbar radiculopathy. While the natural history of the condition remains poorly understand as it typically only comes to clinical attention when significant pseudohypertrophy has occurred and other causes of a swollen limb have been excluded, it is possible that the degree of neural injury required to cause this may be irreversible, or not completely reversible. The value of this diagnosis may lie in providing reassurance to patients and excluding the possibility of more sinister causes

The patient in our case underwent no specific interventions and was given reassurance and encouraged that the etiology of the limb swelling was not an aggressive one. He was referred back to see his spinal surgeon for an opinion regarding repeat surgery, however as he was functionally and symptomatically very well, and there was a possibility that the pseudohypertrophy would not be reversible following repeat surgical exploration, this was decided against.

## Source of funding

There are no financial disclosures.

## Patient consent

Consent provided by patient to Dr John Hynes.
